# Clinically confirmed DEL-1 as a myokine attenuates lipid-induced inflammation and insulin resistance in 3T3-L1 adipocytes via AMPK/HO-1- pathway

**DOI:** 10.1080/21623945.2020.1823140

**Published:** 2020-09-20

**Authors:** Chang Hyuk Kwon, Jaw Long Sun, Myeong Jun Kim, A. M. Abd El-Aty, Ji Hoon Jeong, Tae Woo Jung

**Affiliations:** a Center for Bioinformatics, EONE Laboratories, Incheon, Republic of Korea; b Department of Pharmacology, College of Medicine, Chung-Ang University, Seoul, Republic of Korea; c State Key Laboratory of Biobased Material and Green Papermaking, College of Food Science and Engineering, Qilu University of Technology, Shandong Academy of Science, Jinan, China; d Department of Pharmacology, Faculty of Veterinary Medicine, Cairo University, Giza, Egypt; e Department of Medical Pharmacology, Medical Faculty, Ataturk University, Erzurum, Turkey; f Department of Global Innovative Drugs, Graduate School of Chung-Ang University, Seoul, Republic of Korea

**Keywords:** Myokine, DEL-1, insulin resistance, inflammation, AMPK, HO-1

## Abstract

Regular exercise is the first line of therapy for treating obesity-mediated metabolic disorders, including insulin resistance. It has been reported that developmental endothelial locus-1 (DEL-1) enhances macrophage efferocytosis, resulting in inflammation clearance as well as improves insulin resistance in skeletal muscle. However, the relationship between exercise and DEL-1, and the effects of DEL-1 on insulin signalling in adipocytes have not been fully elucidated to date. Protein expression levels were determined by Western blot analysis. Cells were transfected with small interfering (si) RNA to suppress gene expression. Lipid accumulation levels were detected using Oil red-O staining. Proinflammatory cytokine secretion levels were measured using ELISA. DEL-1 expression levels were induced in the skeletal muscle of people who exercised using microarray analysis. Recombinant DEL-1 augmented AMP-activated protein kinase (AMPK) phosphorylation and haem oxygenase (HO)-1 expression to alleviating inflammation and impairment of insulin signalling in 3T3-L1 adipocytes treated with palmitate. siRNA of AMPK or HO-1 also mitigated the effects of DEL-1 on inflammation and insulin resistance. DEL-1 ameliorates inflammation and insulin resistance in differentiated 3T3-L1 cells via AMPK/HO-1 signalling, suggesting that DEL-1 may be the exercise-mediated therapeutic target for treating insulin resistance and type 2 diabetes.

## Graphical abstract

**Figure:**
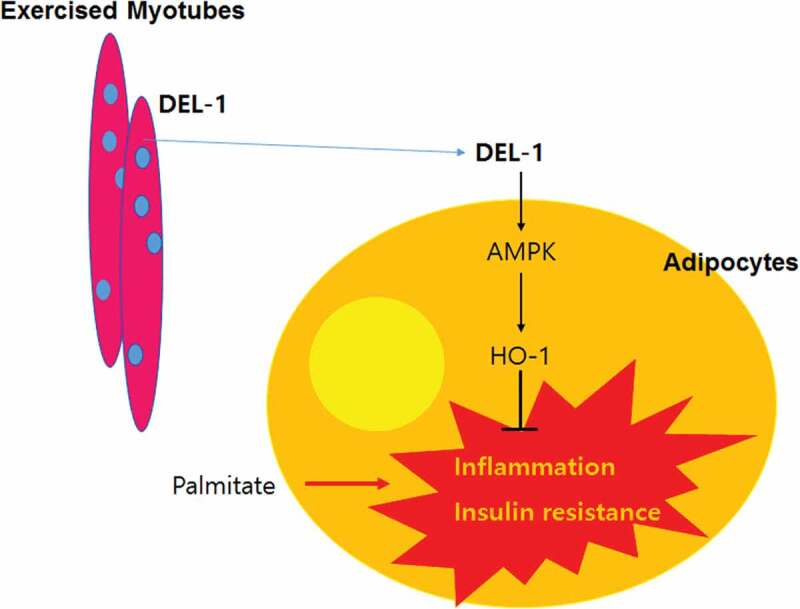

## Introduction

Physical activity has been known as a preferred therapy for treating not only obesity but also obesity-mediated metabolic disorders such as insulin resistance, type 2 diabetes, atherosclerosis and hypertension [1]. Myokines are cytokines or peptides secreted and released by skeletal muscle cells during exercise (muscular contractions) [2]. Various myokines, which increase in muscle cell secretion under exercise conditions, play a crucial role in alleviating diseases associated with metabolic disorders. β-Aminoisobutyric acid (BAIBA) [3] and Meteorin-like protein (METRNL) [4] have been reported to ameliorate inflammation and insulin resistance in skeletal muscle of high-fat diet-fed mice. Gizaw et al. have also reported that transmembrane protein fibronectin type III domain-containing protein 5 (FNDC5)/irisin increases insulin sensitivity and induces β-cell insulin secretion and adipose tissue browning [5]. Furthermore, myocytic fibroblast growth factor 21 (FGF21) also attenuates systemic insulin resistance [6] and atherosclerosis [7] in animal models. Although physical activity remains the preferred strategy for the treatment of metabolic syndrome, exercising consistently can be difficult depending on body conditions, such as age, musculoskeletal conditions and obesity. Therefore, finding novel myokines and thoroughly understanding their functions are crucial in devising new therapeutic strategies for the treatment of diseases associated with metabolic disorders.

Developmental endothelial locus-1 (DEL-1) is a 52-kDa glycoprotein released by vascular endothelial cells during embryological vascular development [8]. Chavakis et al. have reported that DEL-1 with an Arg-Gly-Asp motif in the second EGF domain can be activated through the adhesion of phagocytes and endothelial cells in an autocrine manner [9]. DEL-1 interrupts LFA-1-associated adhesion of leukocytes to endothelial cells, leading to the attenuation of inflammation [10]. Similarly, DEL-1 ameliorates several inflammatory diseases such as pulmonary fibrosis [11], osteoporosis [12] and encephalitis/multiple sclerosis [13]. Contrariwise, interleukin-17 (IL-17), a pro-inflammatory cytokine, downregulates DEL-1 expression in endothelial cells and consequently induces inflammation [12]. Recently, Son et al. reported that DEL-1 augmented AMP-activated protein kinase (AMPK) phosphorylation and ameliorated insulin resistance in mouse skeletal muscle under hyperlipidemic condition [14]. However, to our knowledge, no study has been conducted on the role of DEL-1 in adipocytes.

In this study, we aimed to investigate the effect of exercise on *DEL-1* mRNA expression in human skeletal muscle tissue using microarray and RNA seq analysis to elucidate how exercise can increase insulin sensitivity. Thereafter, we examined the effects of DEL-1 on inflammation and insulin resistance as well as lipid accumulation in 3T3-L1 cells treated with palmitate and explored the underlying DEL-1-mediated mechanism.

## Materials and methods

### Data selection and processing

Microarray: Experiment 1: GSE27285 sample was selected from GEO (Genebank, NCBI) to examine the expression profile of genes expressed in human skeletal muscle tissue. Microarray samples of 39 human skeletal muscle tissues comprising 8 samples of pre-exercise, 16 samples of 3 h post-exercise, and 15 samples of 48 h post-exercise were extracted and analysed using Illumina humanRef-8 v2.0 expression BeadChip. Experiment 2: GSE101931 sample was selected from GEO (NCBI) to examine the expression profile of genes expressed in human skeletal muscle tissue. Microarray samples of 20 human skeletal muscle tissues comprising 5 samples of normal pre-exercise, 5 samples of obese (BMI: 32.1 ± 4) pre-exercise, 5 samples of obese 6 h post-exercise, and 5 samples of obese 24 h post-exercise were extracted and analysed using Illumina humanRef-8 v2.0 expression BeadChip. Experiment 3: GSE101931 sample was selected from GEO (NCBI) to examine the expression profile of genes expressed in human skeletal muscle tissue. Microarray samples of 28 human skeletal muscle tissues comprising 7 samples of normal pre-exercise, 7 samples of type 2 diabetes (T2DM) pre-exercise, 7 samples of T2DM 0 h post-exercise, and 7 samples of T2DM 3 h post-exercise were extracted and analysed using Affymetrix Human Gene 1.0 ST Array [transcript (gene) version].

RNAseq: RNAseq sample, GSE60590, was analysed using 11 samples from the pre-exercise group, and 17 samples from the post-exercise (exercised for 15 min) group. The RNA samples were sequenced as paired-end, 2 × 100 bp on the Illumina HiSeq 2000. A total of 2.43 billion paired-end reads underwent quality and adapter trimming using Trim Galore version 0.2.7. Subsequently, the processed reads were aligned to the human genome reference hg19 with TopHat version 2.0.4 using standard parameters and transcriptome-index on an index built from ENSEMBL v71 transcript annotation. Transcript assembly of the aligned reads was performed using Cufflinks version 2.1.1 and Fragments Per Kilobase of Exon per Million mapped fragments (FPKM) values were calculated using the same software. The aligned reads and the software HTSeq version 0.5.1 were used to count the number of reads per gene. The gene counts generated by HTSeq were further used for differential expression analysis using Limma. Cufflinks gene and isoform FPKM values, which were first corrected for batch effect using ComBat, and used for differential expression analysis. Differential expression of previously unannotated splicing events was performed using Ballgown.

### Cell cultures, reagents and antibodies

The mouse pre-adipocytes 3T3-L1 cell line (ATCC, Manassas, VA, USA) was cultured in Dulbecco’s modified eagle medium (DMEM; Invitrogen, Carlsbad, CA, USA) supplemented with 10% bovine calf serum (Invitrogen), 100 units/mL penicillin and 100 μg/mL streptomycin (Invitrogen). Cells were cultured in a humidified atmosphere of 5% CO_2_ at 37°C. Differentiation was induced 48 h post confluence (2) by cultivation in medium supplemented with 10% foetal bovine serum (FBS, Invitrogen), 1 μM insulin, 0.5 mM IBMX and 0.5 μg/mL dexamethasone for 2 d. This was followed by another 2 d in medium containing 1 μM insulin. Sodium palmitate (Sigma, St Louis, MO, USA) was conjugated to 2% fatty acid-free grade bovine serum albumin (BSA; Sigma) dissolved in DMEM. Differentiated 3T3-L1 cells were treated simultaneously with 200 μM palmitate and 0–1 μM DEL-1 (Aviva System Biology, San Diego, CA, USA) for 24 h after 6 h FBS and insulin starvation. Insulin (10 nM) was used to stimulate insulin signalling (insulin receptor substrate (IRS-1) and Akt) for 3 min. In all experiments, 2% BSA was used as negative control.

### Western blot analysis and antibodies

Differentiated 3T3-L1 cells were harvested and proteins were extracted with lysis buffer (PRO-PREP; Intron Biotechnology, Seoul, Republic of Korea) for 60 min at 4°C. Protein samples (30 μg) were subjected to 10% SDS-PAGE and transferred to a nitrocellulose membrane (Amersham Biosciences, Westborough, MA, USA) and probed with the indicated primary antibodies followed by secondary antibodies conjugated with horseradish peroxidase (Santa Cruz Biotechnology, USA). The signals were detected using enhanced chemiluminescence (ECL) kits (Amersham Biosciences). Anti-phospho Akt (Ser473; 1:1,000), anti-Akt (1:1,000), anti-phospho AMPK (Thr172; 1:1,000), anti-AMPK (1:2,500), anti-phospho NFκBp65 (Ser536; 1:1,000), anti-NFκBp65 (1:2,500), anti-phospho IκB (Ser32; 1:1,000) and anti-IκB (1:1,000) antibodies were purchased from Cell Signalling Technology (Beverly, MA, USA). Anti-phospho IRS-1 (Tyr632; 1:1,000), anti-IRS-1 (1:1,000), anti-HO-1 (1:1,000) and anti-β-actin (1:5,000) were obtained from Santa Cruz Biotechnology.

### Transfection with siRNAs for gene silencing in cells

siRNA oligonucleotides (20 nM) specific for AMPK and HO-1 were purchased from Santa Cruz Biotechnology. To suppress gene expression, cell transfection was performed with Lipofectamine 2000 (Invitrogen) according to the manufacturer’s instructions. In brief, the cells were grown to 60–70% confluence, followed by serum starvation for 12 h after 3T3-L1 cell differentiation. The cells were then transfected with validated siRNA or scramble siRNA at a final concentration of 20 nM in the presence of transfection reagent. After transfection, cells were harvested at 36 h for protein extraction and further analysis.

### Enzyme-linked immunosorbent assay (ELISA)

Serum levels for mouse TNFα and MCP-1 were measured using ELISA kit (R&D Systems, Minneapolis, MN, USA) following the manufacturer’s instructions.

### Measurement of glucose uptake

Glucose uptake levels were measured using Glucose Uptake Assay Kit^TM^ (Abcam, Cambridge, MA, USA). Briefly, proliferating and differentiating 3T3-L1 cells were seeded at a density of 5 × 10^5^ cells/well in black-walled/clear bottom 96-well plates (Corning, Inc., Corning, NY, USA) in DMEM containing 10% FBS. Upon achieving a 95% confluency, differentiation was induced with differentiating media. After 48 h, new media containing either 200 μM palmitate or 0–1 μM DEL-1 was used for additional 10 d. Following treatment, media was removed from wells and treated with 10 nM insulin and 1 mM 2-deoxyglucose (2-DG) for 30 min. Thereafter, plates were centrifuged for 1 min at 500 rpm and incubated for 1 h at 25°C. After 2-DG uptake, the cells were extracted by extraction buffer kit and the uptake levels were measured at a wavelength of OD 412 nm on a BioTek Synergy HT plate reader (BioTek Instruments, Inc., Winooski, VT, USA).

### Statistical analyses

All experiments were performed at least three times. Results are presented as the fold of the highest values (means ± SEM). Student’s *t* test or one-way ANOVA were used to determine statistical significance. All analyses were performed using the SPSS/PC statistical program (version 12.0 for Windows; SPSS, Chicago, IL, USA).

## Results

### DEL-1 gene expression levels in human skeletal muscle were augmented during exercise

**Figure 1.**
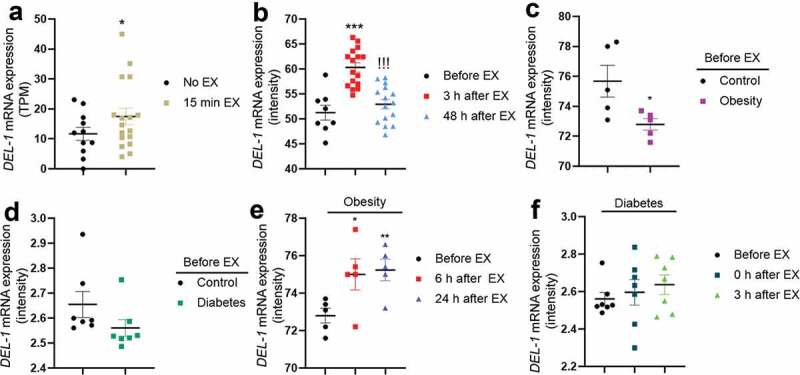
**Exercise induces *DEL-1* gene expression in human skeletal muscle**. (a) Gene expression (TPM; transcripts per million) in resting and exercise for 15 min using RNAseq analysis. (b) gene expression of pre-exercise, 3 h post-exercise and 48 h post-exercise using microarray analysis. Gene expression of normal and obese (c)/diabetic (d) pre-exercise using microarray analysis. (e) gene expression of obese pre-exercise, 6 h post-exercise and 24 h post-exercise using microarray analysis. (f) gene expression of diabetic pre-exercise, 0 h post-exercise and 3 h post-exercise using microarray analysis. ***P < 0.001 and *P < 0.05, when compared to *DEL-1* gene expression in control. ^!!!^*P* < 0.001, when compared to 3 h post-exercise

As DEL-1 has been reported to have several effects (anti-inflammation, anti-insulin resistance and AMPK activation) similar to those of myokine [14], we found that DEL-1 is possibly a myokine. First, RNAseq analysis was performed to confirm the effect of simple exercise on *DEL-1* mRNA expression in skeletal muscle. Based on the results obtained from the two groups (pre- and post-exercise), the expression patterns of *DEL-1* mRNA were confirmed. After exercise, expression of muscle DEL-1 significantly increased by 1.2-fold compared with that in the pre-exercise group (*P* < 0.05) (Figure 1(a)). In order to examine the effects of post-exercise on DEL-1 expression in detail, we set up experimental groups consisting of pre-exercise, 3 h post-exercise and 2 d post-exercise; and the associated microarray data were analysed. We found that *DEL-1* mRNA expression increased at 3 h post-exercise, but decreased to pre-exercise level at 2 d post-exercise, showing that the effects of exercise on DEL-1 expression disappear after 2 d (Figure 1(b)). Decreased *DEL-1* mRNA expression levels were observed in obese/diabetic state (Figure 1(c and d)). However, exercise was likely to increase the expression levels of *DEL-1* mRNA in obese/diabetic state in a time-dependent manner (Figure 1(e and f)).

Taken together, these results suggest that exercise induces *DEL-1* mRNA expression in human skeletal muscle.

### DEL-1 mitigates inflammation and impairment of insulin signalling in adipocytes treated with palmitate

**Figure 2.**
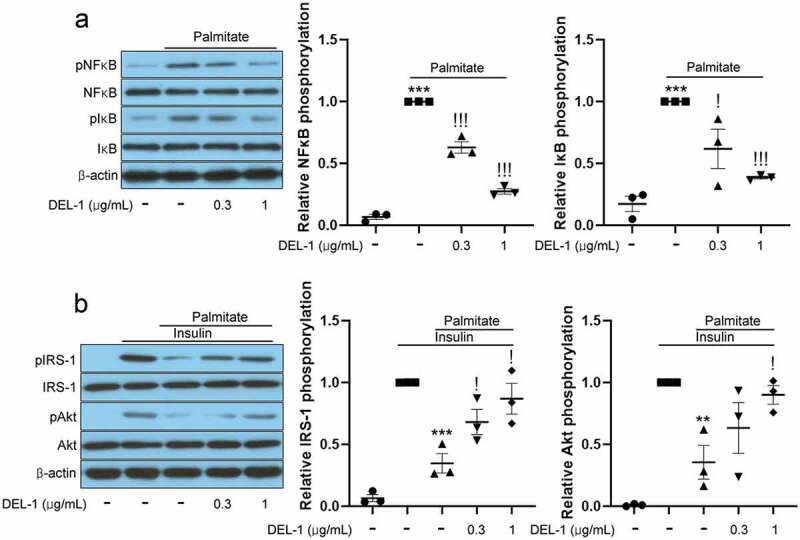
**DEL-1 ameliorates palmitate-induced inflammation and insulin resistance in 3T3-L1 adipocytes**. (a) Western blot analysis of NFκB and IκB phosphorylation in differentiated 3T3-L1 cells treated with palmitate (200 μM) and DEL-1 (0–1 μg/mL) for 24 h. (b) Western blot analysis of IRS-1 and Akt phosphorylation in differentiated 3T3-L1 cells treated with palmitate (200 μM) and DEL-1 (0–1 μg/mL) for 24 h. Human Insulin (10 nM) stimulates IRS-1 and Akt phosphorylation for 3 min. Means ± SEM were obtained from three independent experiments. ***P < 0.001 and **P < 0.01 when compared to control or insulin treatment. ^!!!^*P* < 0.001 and ^!^*P* < 0.05 when compared to palmitate or insulin plus palmitate treatment

On the basis of Figure 1 results, DEL-1, secreted by exercised skeletal muscle, can affect various tissues, including fat tissue, through the bloodstream. Furthermore, inflammation in adipose tissue causes local and systemic insulin resistance [15]. Therefore, we investigated the effects of DEL-1 on inflammation and insulin signalling using recombinant DEL-1 and the differentiated 3T3-L1 cells. Palmitate treatment increased the phosphorylation levels of inflammatory markers such as NFκB and IκB. However, the treatment of 3T3-L1 adipocytes with DEL-1 was found to reverse these changes in a dose-dependent manner (Figure 2(a)). Furthermore, DEL-1 ameliorated palmitate-induced impairment of insulin signalling such as IRS-1 and Akt phosphorylation in a dose-dependent pattern (Figure 2(b)).

### AMPK/HO-1 participates in DEL-1-mediated improvement of inflammation and insulin resistance in adipocytes

AMPK has been reported to resolve inflammation and impairment of insulin signalling in various cell types. Specifically, Sun et al. have reported that DEL-1 actively induced AMPK phosphorylation in mouse skeletal muscle [14]. It has also been documented that HO-1 has an anti-inflammatory property and is regulated by AMPK [16]. Therefore, we further investigated the effects of DEL-1 on AMPK phosphorylation and HO-1 expression. Similar to skeletal muscle, treatment of 3T3-L1 adipocytes with DEL-1 increased AMPK phosphorylation and HO-1 expression in a dose-dependent manner (Figure 3(a)). AMPK siRNA inhibited the expression of DEL-1-induced HO-1, while siRNA-suppressed HO-1 did not alter the phosphorylation DEL-1-induced AMPK (Figure 3(b)). To verify the role of DEL-1 induced AMPK and HO-1, we suppressed AMPK and HO-1 expression using respective siRNA. Knockdown of AMPK or HO-1 using siRNA mitigated the suppressive effects of DEL-1 on the palmitate-induced NFκB and IκB phosphorylation as well as the secretion of proinflammatory cytokines such as TNFα and MCP-1 (Figure 3(c-e)). Furthermore, siRNA-mediated AMPK or HO-1 expression abrogated the effects of DEL-1 on the palmitate-induced impairment of insulin-stimulated IRS-1 and Akt phosphorylation and glucose uptake in 3T3-L1 cells (Figure 3(f)).

**Figure 3.**
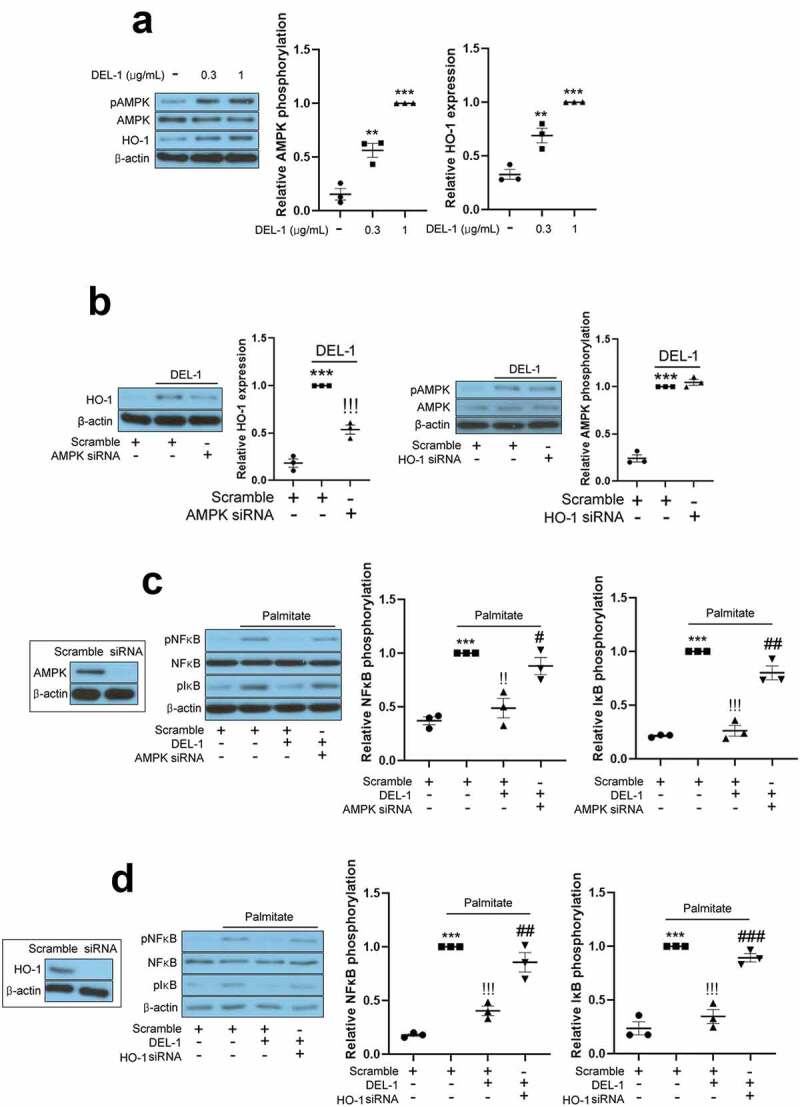
**AMPK/HO-1 contributes to the attenuation of inflammation and insulin resistance in 3T3-L1 adipocytes**. (a) Western blot analysis of AMPK phosphorylation and HO-1 expression in differentiated 3T3-L1 cells treated with DEL-1 (0–1 μg/mL) for 24 h. (b) Western blot analysis of AMPK phosphorylation and HO-1 expression in AMPK or HO-1 siRNA-transfected 3T3-L1 adipocytes treated with DEL-1 (1 μg/mL) for 24 h. Western blot analysis of NFκB and IκB phosphorylation in AMPK (c) or HO-1 (d) siRNA-transfected 3T3-L1 myocytes treated with palmitate (200 μM) and DEL-1 (1 μg/mL) for 24 h. (e) ELISA for TNFα and MCP-1 release by AMPK or HO-1 siRNA-transfected 3T3-L1 adipocytes treated with DEL-1 (1 μg/mL) for 24 h. Western blot analysis of phosphorylation of IRS-1 and Akt and glucose uptake measurement (f) in AMPK or HO-1 siRNA-transfected 3T3-L1 adipocytes treated with 200 μM palmitate and DEL-1 (1 μg/mL) for 24 h. Human insulin (10 nM) stimulates insulin signalling for 3 min. Means ± SEM were obtained from three independent experiments. ***P < 0.001 when compared to control or insulin treatment. ^!!!^*P* < 0.001, ^!!^*P* < 0.01 and ^!^*P* < 0.05 when compared to palmitate or insulin plus palmitate treatment. ^###^*P* < 0.001, ^##^*P* < 0.01 and ^#^*P* < 0.05 when compared to the insulin, palmitate plus DEL-1 or insulin, palmitate plus DEL-1 treatment

**Figure 3.**
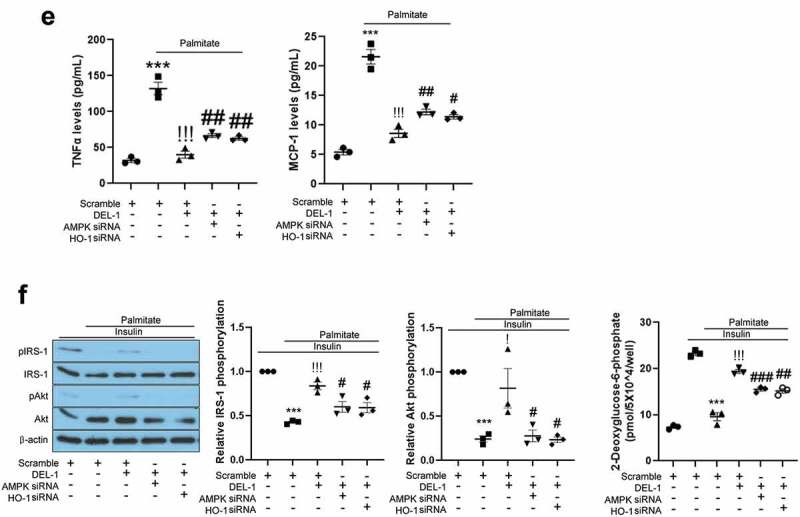
(Continued)

### DEL-1 did not affect intracellular lipid accumulation, although it enhanced thermogenesis in 3T3-L1 cells

**Figure 4.**
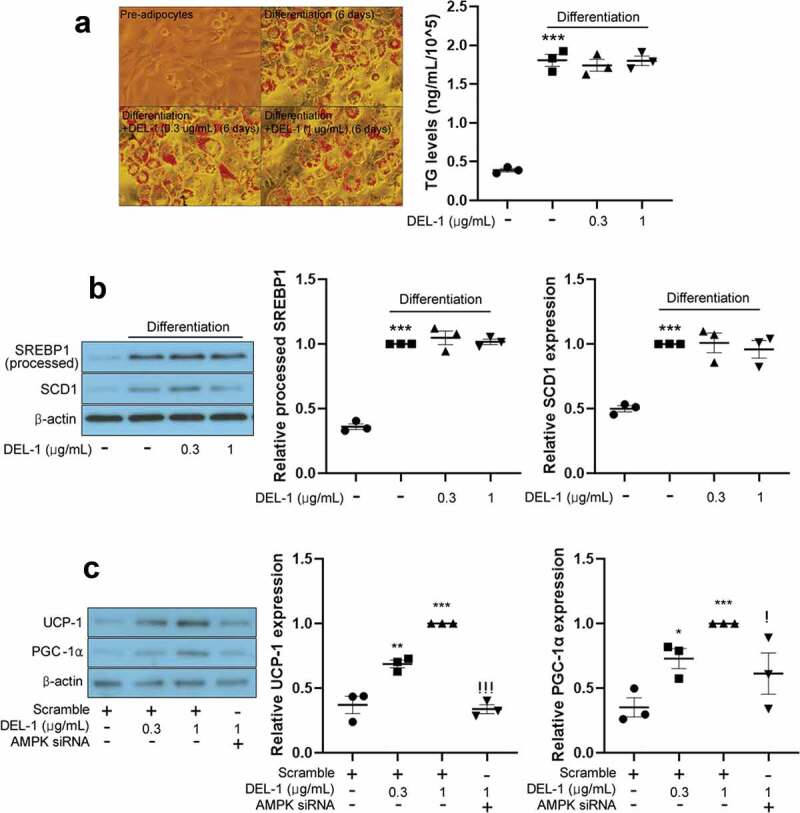
**DEL-1 did not affect 3T3-L1 differentiation**. (a) Oil-red O staining in differentiated 3T3-L1 cells in the presence of DEL-1 (0–1 μg/mL) for 6 d. TG accumulation was quantitated by modified TG assay kit. (b) Western blot analysis of processed SREBP1 and SCD1 expression in 3T3-L1 adipocytes treated with DEL-1 (0–1 μg/mL) for 6 d. (c) Western blot analysis of UCP-1 and PGC-1α expression in AMPK siRNA-transfected 3T3-L1 adipocytes treated with DEL-1 (0–1 μg/mL) for 24 h. Means ± SEM were obtained from three independent experiments. ***P < 0.001 and **P < 0.01 when compared to control. ^!^*P* < 0.05 when compared to DEL-1 (1 μg/mL) treatment

Myokines activate AMPKs such as BAIBA [3,17], FGF21 [18] and METRNL [4] to cause body weight loss in animal models. To examine the effect of DEL-1 on adipocyte differentiation, intracellular lipids were stained by Oil Red-O and quantified. To our surprise, even at a concentration of 1 μg/mL, DEL-1 not only improved inflammation and insulin resistance but also upregulated AMPK phosphorylation. However, lipid accumulation (Figure 4(a)) and lipogenic gene expression (Figure 4(b)) were not changed in DEL-1-treated 3T3-L1 adipocytes. Impairment of thermogenesis promotes obesity and insulin resistance [19]. Therefore, we further examined the effect of DEL-1 on thermogenesis in 3T3-L1. Treatment of 3T3-L1 adipocytes with DEL-1 augmented thermogenic markers, such as UCP-1 and PGC-1α expression in a dose-dependent way. However, AMPK siRNA mitigated DEL-1 effect (Figure 4(c)).

## Discussion

Obesity-induced chronic inflammation has been reported to play a causative role in the development of metabolic disorders including insulin resistance [20]. Specifically, inflammation in adipose tissue contributes to the development of systemic insulin resistance [21]. Adipose tissue secretes a variety of hormones, cytokines and metabolites (termed as adipokines) that are critical for maintaining cellular metabolic homoeostasis via regulating insulin signalling through autocrine and paracrine manners [22]. Therefore, it is of vital importance to effectively suppress adipose tissue inflammation caused by obesity.

There are various reports of the effects of regular physical activity on systemic inflammation and insulin resistance. For example, regular exercise could reduce fat mass and attenuate inflammation in adipose tissue to prevent further systemic inflammation [23]. Exercise suppresses the production of TNFα, which is known to cause insulin resistance through skeletal muscle IL-6 secretion without weight loss while inducing secretion of anti-inflammatory cytokines to improve systemic inflammation [24]. Furthermore, exercise activates AMPK in various organs, including skeletal muscle. Therefore, as the skeletal muscle is the organ most involved in exercise, substances secreted during exercise should be identified to effectively activate AMPK. Firstly, we verified DEL-1 as a myokine secreted by skeletal muscle during exercise using RNA seq and microarray data analysis. In this study, we found that exercise enhanced, or tended to increase the expression levels of *DEL-1* mRNA in human skeletal muscle. Furthermore, impaired *DEL-1* mRNA expression levels were detected in both obese and T2DM groups. These clinical results provide a reason why DEL-1 as a myokine can be used to treat obesity-mediated metabolic disorders, including diabetes. Next, our result and a previous report [14] led us to examine the effects of DEL-1 on lipid-induced inflammation and the aggravation of insulin signalling in adipocytes. In this study, we found that DEL-1 treatment alleviated inflammation and insulin resistance in adipocytes treated with palmitate.

AMPK plays a central role in energy homoeostasis and intracellular sensing of ATP consumption associated with energy metabolism in skeletal muscle [25]. AMPK stimulates the production of ATP and catabolism in glucose and fat oxidation [26]. Thus, for a long time, AMPK has been attracting attention as a therapeutic target for diseases associated with metabolic disorders, and many studies are still ongoing. Activation of AMPK ameliorates lipid-induced insulin resistance in tissues such as skeletal muscle [27], liver [28] and adipose tissues [29] through various pathways. Metabolic disorders are usually presented as low-grade chronic inflammation primarily in adipose tissue [30]. Accumulating reports have indicated that the AMPK-dependent pathway suppresses the NFκB-induced inflammatory responses whereas impaired AMPK activity contributes to the increase in inflammation. Additionally, a plethora of evidences have shown that an analogue of adenosine monophosphate (AICAR), functions as an activator of AMPK, attenuates the progression of inflammatory diseases such as acute and chronic colitis [31], acute injury in lung [32], hepatic injury and fibrosis [33], and autoimmune encephalomyelitis [34] in murine models. Furthermore, an AMPK activator metformin ameliorates systemic inflammation through inhibiting C-reactive protein and IL-6 in patients with metabolic syndrome [35]. Jung et al. have reported that myokine BAIBA alleviates LPS-induced inflammatory responses in HUVEC and THP-1 cells through the AMPK-mediated pathway [36]. Moreover, myokine fibronectin type III domain containing 4 (FNDC4) ameliorates lipid-induced inflammation and insulin resistance in adipocytes through AMPK-dependent signalling [16]. In the present study, we found that DEL-1 augmented AMPK phosphorylation in 3T3-L1 adipocytes. siRNA-mediated AMPK suppression mitigated the suppressive effects of DEL-1 on palmitate-induced inflammation and insulin resistance. These results suggest that DEL-1-mediated AMPK activation contributes to the improvement of inflammation and attenuation of insulin resistance in adipocytes. In order to explore how DEL-1 increases AMPK phosphorylation, further studies are required to identify DEL-1 selective receptors.

Haem oxygenase (HO) is a rate-limiting enzyme for haem degradation and carbon monoxide (CO), iron and biliverdin production, which is converted into bilirubin via biliverdin reductase-mediated signalling [37]. Two isoforms of haem oxygenase, HO-1 and HO-2, have been identified. HO-2 is constitutively expressed in the brain and testis [38], whereas HO-1 is basally expressed in many cell types and tissues. HO-1 is highly induced by several cellular stimuli such as oxidative stress [39], ER stress [40] and inflammation [41]. These properties of HO-1 are closely related to cytoprotective effects from stimuli. In particular, the anti-inflammatory functions of HO-1 have been documented through various studies. Alcaraz et al. reviewed that HO-1-mediated signalling or – derived products, such as CO plays a pivotal role in the inhibition of inflammatory responses [42]. Paine et al. emphasized that pharmacological upregulation of HO-1 is a therapeutic strategy for treating inflammatory diseases [43]. Adenoviral overexpression of HO-1 improves TNFα-mediated airway inflammation through the suppression of oxidative stress [44]. HO-1 transgenic mice have demonstrated anti-inflammatory effects against LPS-mediated inflammation [45]. Recently, Lee et al. reported that kynurenic acid-induced HO-1 inhibits LPS-stimulated inflammatory responses in endothelial cells [46]. Additionally, Jung et al. demonstrated that salsalate alleviates atherosclerotic responses via HO-1-dependent suppression of inflammation [47]. Myokine FNDC4 suppresses palmitate-induced inflammation through the upregulation of HO-1 expression in 3T3-L1 adipocytes [16]. Conversely, high severity of chronic inflammation and sepsis caused by endotoxin is observed in HO-1 knockout mice [48]. Furthermore, innate and adaptive immune responses are significantly induced in HO-1-deficient mice [49,50]. In the current study, we examined the effects of DEL-1 on HO-1 expression in 3T3-L1 cells. Treatment of 3T3-L1 adipocytes with DEL-1 increased HO-1 expression. We next examined the role of DEL-1-induced HO-1 in inflammation and insulin signalling in 3T3-L1 adipocytes treated with palmitate. HO-1 siRNA mitigated the effects of DEL-1 on palmitate-induced inflammatory markers as well as the aggravation of insulin signalling. Furthermore, AMPK siRNA abrogated the effect of DEL-1 on HO-1 expression whereas siRNA-suppressed HO-1 did not have any influence on AMPK phosphorylation in DEL-1-treated 3T3-L1 adipocytes. These results suggest that AMPK-regulated HO-1 by DEL-1 induces improvement of palmitate-mediated inflammation and insulin signalling impairment.

Because AMPK activation suppresses lipid accumulation in adipocytes [51], herein we found that DEL-1 treatment augmented expression of thermogenic markers via AMPK signalling, and we further examined whether DEL-1 affects lipid accumulation in 3T3-L1 adipocytes during differentiation. Our results show that lipid accumulation and lipogenesis in 3T3-L1 adipocytes were not changed by DEL-1 at 1 μg/mL, suggesting that a concentration of 1 μg/mL DEL-1 was not sufficient to inhibit lipid accumulation in adipocytes. Therefore, further studies should focus on the treatment of DEL-1 at higher concentrations or extended treatment time.

One of the study limitations is that the *in vitro* data do not indicate that the observed effect is specific to the exercised skeletal muscle DEL-1. Therefore, further studies, such as co-culture skeletal muscle cells with adipocytes or to treat the supernatants of ‘exercised-like’ skeletal muscle cell with adipocytes, are needed to understand this mechanism.

**Figure 5.**
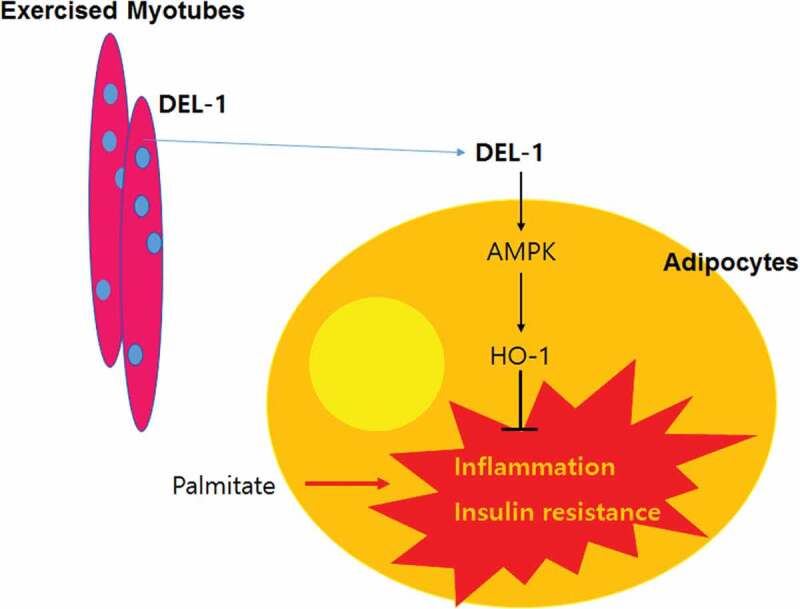
Schematic diagram for the effects of DEL-1 on inflammation and insulin resistance in adipocytes

In conclusion, we have identified DEL-1 as an exercise-induced myokine. Hence, we have demonstrated that DEL-1 alleviates lipid-induced insulin resistance in adipocytes through AMPK/HO-1 axis-mediated suppression of inflammation (Figure 5). Pharmacologic activation of AMPK/HO-1 signalling by myokine DEL-1 may be an effective therapeutic approach for treating systemic insulin resistance and type 2 diabetes.
